# Biochemical testing for inborn errors of metabolism: experience from a large tertiary neonatal centre

**DOI:** 10.1007/s00431-022-04588-4

**Published:** 2022-08-10

**Authors:** Esme Dunne, Daniel O’Reilly, Claire A. Murphy, Caoimhe Howard, Grainne Kelleher, Thomas Suttie, Michael A. Boyle, Jennifer J. Brady, Ina Knerr, Afif El Khuffash

**Affiliations:** 1 Department of Paediatrics, Rotunda Hospital, Dublin 1, Ireland; 2 Department of Paediatrics, RCSI, Dublin 2, Ireland; 3 National Centre for Inherited Metabolic Disorders, Children’s Health Ireland at Temple Street, Dublin 1, Ireland; 4 Department of Laboratory Medicine, Rotunda Hospital, Dublin 1, Ireland; 5 Department of Clinical Biochemistry, Children’s Health Ireland at Temple Street, Dublin 1, Ireland; 6 University College Dublin School of Medicine, Belfield, Dublin 4, Ireland

**Keywords:** Inborn errors of metabolism, Neonates, ICU, Diagnostics

## Abstract

Inborn errors of metabolism are an individually rare but collectively significant cause of mortality and morbidity in the neonatal period. They are identified by either newborn screening programmes or clinician-initiated targeted biochemical screening. This study examines the relative contribution of these two methods to the identification of inborn errors of metabolism and describes the incidence of these conditions in a large, tertiary, neonatal unit. We also examined which factors could impact the reliability of metabolic testing in this cohort. This is a retrospective, single-site study examining infants in whom a targeted metabolic investigation was performed from January 2018 to December 2020 inclusive. Data was also provided by the national newborn screening laboratory regarding newborn screening diagnoses. Two hundred and four newborns received a clinician-initiated metabolic screen during the time period examined with 5 newborns being diagnosed with an inborn error of metabolism (IEM) (2.4%). Of the 25,240 infants born in the hospital during the period examined, a further 11 newborns had an inborn error of metabolism diagnosed on newborn screening. This produced an incidence in our unit over the time described of 6.34 per 10,000 births. This number reflects a minimum estimate, given that the conditions diagnosed refer to early-onset disorders and distinctive categories of IEM only. Efficiency of the clinician-initiated metabolic screening process was also examined. The only statistically significant variable in requiring repeat metabolic screening was early day of life (*z*-score = − 2.58, *p* = 0.0098). A total of 28.4% was missing one of three key metabolic investigation parameters of blood glucose, ammonia or lactate concentration with ammonia the most common investigation missing. While hypoglycemia was the most common clinical rationale for a clinician-initiated metabolic test, it was a poor predictor of inborn error of metabolism with no newborns of 25 screened were diagnosed with a metabolic disorder.

*Conclusion*: Clinician-targeted metabolic screening had a high diagnostic yield given the relatively low prevalence of inborn errors of metabolism in the general population. Thoughts should be given to the rationale behind each targeted metabolic test and what specific metabolic disease or category of inborn error of metabolism they are concerned along with commencing targeted testing.

**Table:** 

**What is Known:** *• Inborn errors of metabolism are a rare but potentially treatable cause of newborn mortality and morbidity.* *• A previous study conducted in a tertiary unit in an area with limited newborn screening demonstrated a diagnostic yield of 5.4%.*	
**What is New:** *• Clinician-initiated targeted metabolic screening has a good diagnostic performance even with a more expanded newborn screening programme.* *• Further optimisation could be achieved by examining the best timing and also the rationale of metabolic testing in the newborn period.*

**What is Known:**

*• Inborn errors of metabolism are a rare but potentially treatable cause of newborn mortality and morbidity.*

*• A previous study conducted in a tertiary unit in an area with limited newborn screening demonstrated a diagnostic yield of 5.4%.*

**What is New:**

*• Clinician-initiated targeted metabolic screening has a good diagnostic performance even with a more expanded newborn screening programme.*

*• Further optimisation could be achieved by examining the best timing and also the rationale of metabolic testing in the newborn period.*

## Introduction

Inborn errors of metabolism (IEM) are an individually rare but collectively significant cause of illness in the neonatal period with a collective incidence of between 1.5 and 4 per 10,000 live births [1]. They are heterogeneous group with over 1000 disease entities described which affect different components of the metabolic pathway and may not be immediately apparent in the neonatal period [2]. Given this heterogeneity, and their individual rarity, many clinicians attempt to utilise a targeted screen for a large proportion of them using a combination of biochemical tests (urea and electrolytes, liver function tests, blood gases, blood glucose concentrations, plasma ammonia) and a selection of second-line metabolic investigations (plasma amino acids, acylcarnitine profile, urinary organic acids and urinary glycosaminoglycans, GAGs) before proceeding to specific metabolic tests (for example very long chain fatty acids, lysosomal enzymes, transferrin isoforms) [3, 4]. In principle, this is a rational approach; however, caution must be maintained as toxic metabolites often require time to accumulate and testing too early may result in an indeterminate value and require repeat testing. Additionally for some conditions, a narrow spectrum of tests could be pursued and achieve as good if not better diagnostic accuracy given the relatively low pre-test probability of an IEM in the general population, albeit higher in symptomatic individuals.

In addition to diagnostic testing of a symptomatic newborn, in many jurisdictions, there exists a newborn screening programme which typically use dried blood spot cards to screen for the presence of a number of IEM and other genetic conditions (such as congenital hypothyroidism, cystic fibrosis, sickle cell disease). In Ireland, 8 conditions are included of which 6 are IEM; phenylketonuria (PKU), maple syrup urine disease (MSUD), classical galactosemia, classical homocystinuria (HCU), glutaric aciduria type 1 (GA), and medium-chain acyl-CoA dehydrogenase deficiency (MCADD) [5, 6]. There is also positive selection of neonates from a traveller ethnicity for a Beutler test for galactose-1-phosphate uridyltransferase (GALT) activity as part of their newborn screening process, due to a high rate of classical galactosemia in this population [7]. The number of conditions screened is comparable to neighbouring countries such as the UK, France and Switzerland (9, 6 and 10 conditions included respectively) but is less than other European nations such as Sweden, Germany and Finland (25, 17 and 21 conditions included respectively) [8]. While there is scope to expand newborn screening in Ireland, this is still in a preclinical phase at present.

A study conducted in a large centre in Paris examined the diagnostic contribution of targeted metabolic testing in infants admitted to the NICU from 2010 to 2014 demonstrated from 196 neonates; 11 infants had an IEM identified (5.6% of the target population) [9]. Additionally, every infant included was sufficiently unwell enough to be admitted to neonatal intensive care.

New diagnostic technologies are rapidly evolving for the care of the undifferentiated critically unwell infant or neonate including rapid exome sequencing and genetic panels which can definitively identify genetic causes of disease in the rapidly deteriorating child in as little as 4 days [10]. Given the large volume of blood required to perform a series of metabolic investigations and the difficulty in successful phlebotomy in the neonatal population, there is a proportion of neonates who do not get a full biochemical and metabolic assessment who may benefit from the use of newer technologies.

The purpose of this study therefore was to establish the diagnostic yield of biochemical testing and its role in establishing a definitive diagnosis in the unwell neonate. Our hypothesis was that biochemical testing will have a poorer diagnostic yield in the context of a wider newborn screening programme. We also examined what factors determined the requirement for continued biochemical monitoring.

## Methods

### Study population

This is a single-site retrospective observational study of a large, tertiary neonatal unit with approximately 8000 deliveries per year from January 2018 to December 2020 inclusive. The total number of deliveries eligible for newborn bloodspot screening was 25,240 infants. Newborn bloodspot screening in Ireland is performed on dried bloodspots taken between 72 and 120 h of age. This is performed using liquid chromatography tandem mass spectrometry (LC–MS/MS). Information regarding the number of children born on site (the Rotunda hospital) and those diagnosed with an IEM based on newborn screening was provided by the National Newborn Bloodspot Screening Laboratory.

Additionally, we examined electronic health records for any infant where a diagnostic assessment of IEM was undertaken through the ordering of either (i) plasma ammonia or (ii) plasma amino acids over this study period. Plasma amino acids were measured using ion exchange chromatography. Other metabolic investigations ordered in this cohort included dried bloodspot acylcarnitines which were measured using LC–MS/MS and urine organic acids/GAGs which were measured using gas chromatography mass spectroscopy (GCMS). Infants who received an investigation as a follow-up to a positive newborn screen were excluded.

### Data collection

Data was collected retrospectively on the above cohort of newborns through laboratory records regarding investigations undertaken. Each child who had a plasma ammonia or plasma amino acid performed had their chart examined. Each child had their demographic data (corrected gestational age, age in days when metabolic investigations commenced), clinical data (rationale for testing, diagnosis if established by discharge, total parenteral nutrition at time of investigations, total parenteral nutrition at any point prior to metabolic investigations), biochemical data (presence or absence of a metabolic acidosis, highest recorded lactate, highest recorded ammonia, lowest recorded blood glucose level) and data regarding metabolic investigations (plasma amino acids, urine organic acids, urinary glycosaminoglycans, acylcarnitine profile, any other metabolic investigations requested, repeat metabolic investigations ordered).

### Statistical analysis

Descriptive statistics are reported for the majority of findings given the overall low numbers involved. To examine which clinical features increased, the likelihood of an infant requiring repeat investigations a logistic regression was performed using R (version 4.1.1, Computing, Vienna, Austria. URL http://www.R-project.org/). Included variables were those suspected to increase the likelihood of an inadequate first metabolic investigations. These were corrected: gestational age, age in days, total parenteral nutrition when the metabolic investigations took place and total parental nutrition at any point prior to testing. A *p*-value > 0.05 was considered significant.

### Ethical considerations

This study was approved by the local research and ethics committee at the Rotunda hospital (reference number RAG 2021 005). Patient data was handled in accordance with good clinical practice throughout. Minimisation of data was felt to be crucial in maintaining anonymity of included subjects in accordance with general data protection regulation; therefore, only data which was felt to materially impact on laboratory testing was held, i.e., information on sex and ethnicity was not included.

## Results

### Age demographics of children undergoing diagnostic evaluation for IEM

A total of 204 neonates had a diagnostic assessment for the presence of an IEM during the study period. They had median corrected gestational age of 37 weeks and 5 days (range 23 and 3–41 and 6 days). Testing occurred at a median day of life of day 4 (range 1–180 days).

### Presenting findings and clinical presentations

Over the time period investigated, there were 5 infants with IEMs identified in the population who had a series of metabolic investigations performed (*n* = 204). These included 1 neonate with a urea cycle defect, 2 infants with lysosomal storage disorders (mucolipidosis type II, mucopolysaccharidosis type 1), 1 infant with a disorder of peroxisome biogenesis and 1 infant with a mitochondrial disorder. This represents 2.4% of the cohort who underwent metabolic diagnostic tests.

**Fig. 1:**
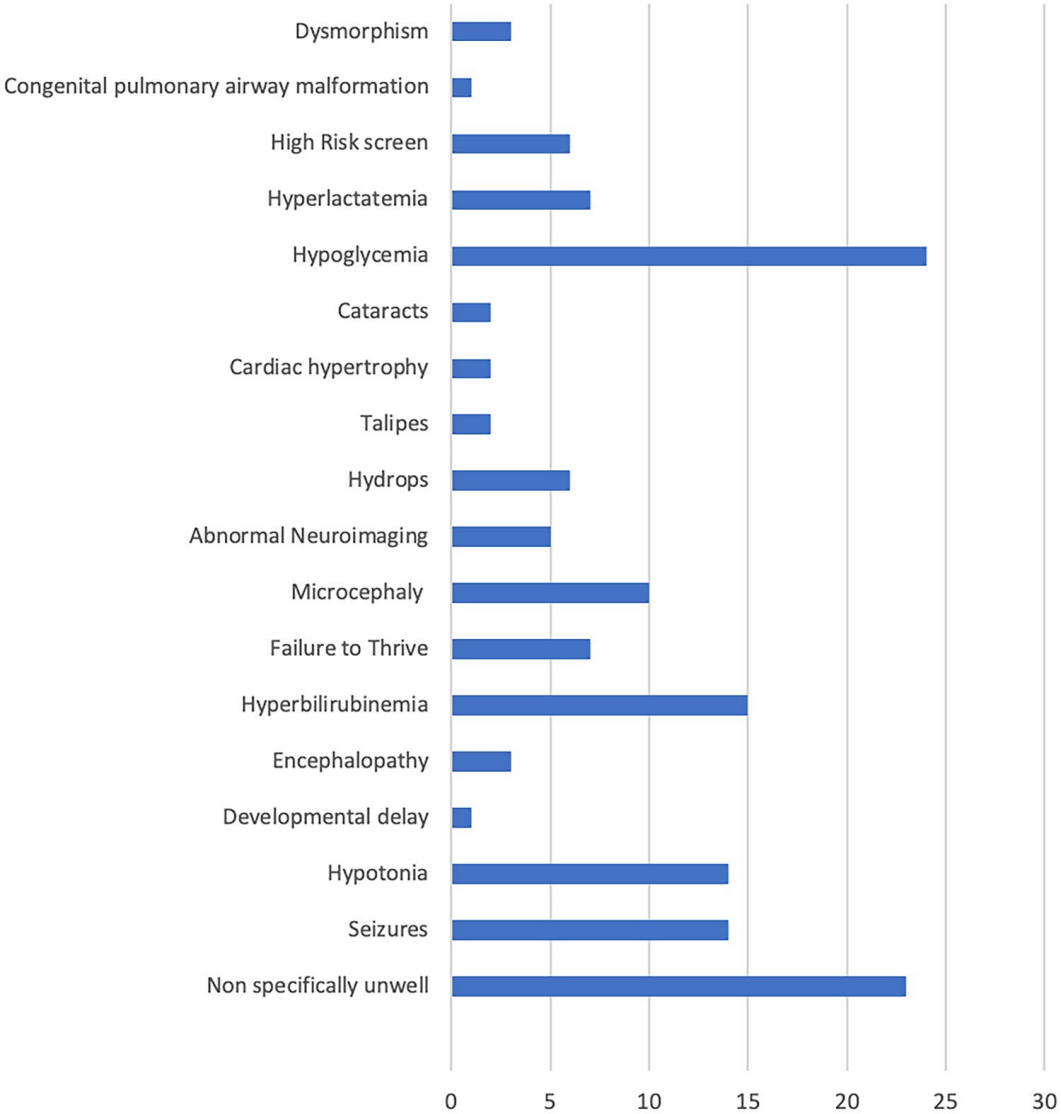
Graph illustrating most common symptoms for which children had a targeted metabolic workup. Forty-one children had > 1 clinical indication (total number of infants included, *n* = 204)

The most common rationale for testing was hypoglycaemia (*n* = 25), followed by the non-specifically unwell infant (*n* = 23) and prolonged hyperbilirubinemia (*n* = 15) (Fig. 1). Both children with lysosomal storage disorders were investigated as a high-risk screen, i.e., a family member had previously presented with the condition. The infant with mucolipidosis type II was also identified as dysmorphic. The infant with a disorder of peroxisomal biogenesis was identified as both dysmorphic and having a seizure in the neonatal period. The remaining diagnoses (UCD, mitochondrial disorder) were made in children who were non-specifically unwell.

**Fig. 2:**
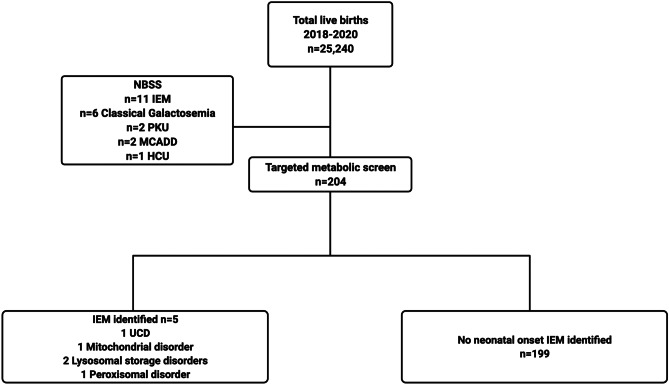
Flow diagram illustrating diagnostic yield of newborn screen in combination with a targeted metabolic screen

A further 11 neonates were identified by the newborn screening programme with an IEM. Of these children, 6 were identified as having classical galactosemia, 2 had PKU, 2 had MCADD and a single child was identified as having HCU (Fig. 2).

A total of 16 infants were identified as having an early-onset IEM identifiable through the biochemical tests performed through this single site producing a minimum incidence of 6.34 per 10,000 births.

### Data completeness

To examine how many children had full biochemical assessment prior to metabolic testing, 3 testing parameters which were felt to be key to the diagnosis of IEM in the intermediary metabolic pathways were examined: (i) blood/plasma glucose concentration (mmol/l) measured by point of care testing or in the laboratory, (ii) plasma ammonia level (μmol/l) and (iii) plasma lactate (mmol/l) (Table 1).

**Table 1:** Summary of missing data regarding first line biochemical testing for IEM

Biochemical test (standardised units)	Median concentration (range)	Number of infants with no recorded result (%)
Glucose (mmol/l)	3.1 (0.2–5.7)	13 (6.3)
Lactate (mmol/l)	2.69 (0.9–20)	27 (13.2)
Ammonia (µmol/l)	63 (28–629)	37 (18.3)
Missing 1 or more of glucose, lactate or ammonia		58 (28.4)

### Number of infants requiring continued biochemical monitoring

Given the high risk of anaemia and difficulty in reliable phlebotomy in this group, we examined how many infants required repeat sampling and further monitoring. In total, *n* = 81 infants needed a test repeated representing 39.7% of this cohort. Of these tests, *n* = 65 infants required a repeat blood sample representing 31.9% of the cohort.

### Risks for requiring continued biochemical monitoring

Lower gestational age and presence of total parenteral nutrition at time of testing trended towards a higher risk of repeat sampling in a non-statistically significant manner (cGA *z*-score = − 1.337, *p* = 0.18; TPN at time of testing *z*-score = 1.019, *p* = 0.3). Any prior TPN was not associated with an increased risk of testing (*z*-score = 0.758, *p* = 0.45).

Day of life correlated in a statistically significant way to repeat sampling (*z*-score = − 2.58, *p* = 0.0098) even if a Bonferroni correction was applied to a significance level of *p* = 0.05 (*p* = 0.05/4, *p* = 0.0125).

## Discussion

The purpose of this study was to examine the utility and clinician performance of a series of commonly used metabolic investigations in a tertiary neonatal unit. Investigation for IEM in the critically unwell infant is of foremost importance for several reasons. Firstly, if proper and disease-specific treatment can be instituted, possible long-term injury or early death can be avoided in affected individuals. Secondly, to inform prognostication for parents in the case of infants for whom no curative therapy is available. Thirdly, to avoid an inaccurate treatment algorithm being applied to a critically unwell infant (i.e., escalation for therapies for sepsis or renal failure) [3].

Between NBSS (newborn bloodspot screening) and metabolic investigations, a number of metabolic diagnoses were made over the 3-year period. The estimated incidence of IEM in Ireland is 1.5–4 per 10,000 births while in our cohort, the incidence rate was 6.34 per 10,000 births, a relative 1.5–fourfold increase on baseline estimates. There are a number of reasons this may be the case. The estimated incidence for IEM in Ireland is based on a study performed in British Columbia which included a predominantly Caucasian population [1]. While the Irish cohort would also be predominantly Caucasian and would have a similar size of population (approx. 5 million individuals), a number of differences would be expected to exist. For example, Irish travellers are an endogamous, ethnically Irish population numbering around 40,000 with a known higher rate of IEM [11]. This along with other founder effects in the Irish population may contribute to the higher incidence of IEM than predicted by the British Columbia study.

These data were collected in a tertiary neonatal unit which caters for high risk pregnancies for a large proportion of the Irish population which may include families at risk for IEM. While high-risk screens only contributed to a small number of metabolic investigations undertaken clinically (*n* = 6), women who had previously had a child identified by newborn screening may subsequently have chosen to deliver in a higher acuity unit which then led to more infants being identified on NBSS than would be expected.

While only 2.4% of targeted metabolic screens demonstrated the presence of an IEM, this is a large diagnostic yield for conditions with an incidence approaching 1.5–4 per 100,000 births. While 3 of the conditions identified clinically are likely to have specific clinical features (mucopolysaccharidoses (*n* = 2), peroxisomal biogenesis defects), the remaining two conditions (urea cycle disorder, mitochondrial disorder) identified would be unlikely to be identified purely on first line biochemical testing. This underlines the value of properly performed metabolic testing in uncovering a serious metabolic disease.

A large number of metabolic investigations required repeat or were of limited diagnostic value when first taken. A large number of these were taken at a very early stage prior to the build-up of sufficient metabolites for analysis [4]. This may favour the use of newer quick turnaround genetic tests which are not dependent on the presence of biochemical derangements especially as these tests are now becoming more cost-effective. For clinicians in neonatal units, the desire to screen for IEM early, and therefore institute appropriate treatment, should be balanced against the requirement in many of these conditions for metabolites to accrue for detection (albeit with a number of notable and important exceptions such as non-ketotic hyperglycinaemia, glutaric aciduria type II and primary lactic acidosis) [4]. Early consultation with clinicians specialised in paediatric metabolic medicine may aid in this decision making process; however, access may be limited and existing services under pressure in different clinical settings. Expanded biochemical testing together with targeted genetic analysis may enable the diagnosis, and clinicians should be vigilant of significant clinical, genetic and biochemical variability of IEM which warrants thorough investigation when suspicion arises [12].

A number of symptom complexes dominate the number of tests which were performed but did not produce a single positive diagnosis. For example, 25 infants were investigated for hypoglycaemia either as the sole clinical factor or in conjunction with others. Given the likely aetiology of this symptom is hyperinsulinemia (particularly in preterm infants), a stepped approach may be warranted prior to a full battery of metabolic investigations with a very low likelihood of uncovering an IEM particularly in an infant with a high glucose infusion rate (GIR) [4].

A significant proportion of data for each infant was incomplete which is likely due to a combination of factors. When performing targeted metabolic tests, a number of blood tests are performed simultaneously with what is often a difficult blood sample to obtain. By the time, it becomes clear that a blood sample is insufficient or missing, and the clinical picture may have evolved to suggest an alternative pathological basis for their condition. Some routine collected point of care data may not simply have been recorded on the EHR (e.g., lactate and glucose measurements which were taken as part of a blood gas measurement). Finally, while the electronically generated order set is created in our unit, it does not include elements such as ammonia, which needs to be separately requested by the clinician. A computer-based solution which enables targeted biochemical testing, including glucose, lactate and ammonia, in acutely ill neonates where sepsis is suspected and urgent blood tests are requested, could promote early identification of IEM patients with disorders such as urea cycle defects, organic acidaemia or mitochondrial disorders.

The early use of genomic sequencing strategies in the critically unwell neonate has shown promise in a number of studies [13, 14]. While there have been impressive diagnostic yields demonstrated in studies to date, cost-effectiveness has only been demonstrated in a PICU and not a NICU population [15]. Ongoing trials as part of the Newborn Sequencing in Genomic Medicine and Public Health (NSIGHT) consortium are being performed to establish the benefits of genomic sequencing in newborns as well as the cost effectiveness of such a strategy [16]. Ultimately, a combination of biochemical and genetic screening may be the most useful diagnostic strategy in the context of IEM.

This study highlights the additional diagnostic yield which can be achieved through biochemical testing for IEM even in the context of a wider NBSS and also has a number of limitations. Biochemical testing protocols for investigation of IEM can vary from institution to institution and the experience on our single site may not be exactly the same as others especially in countries with an even wider spectrum of metabolic disease examined by NBSS, or in sites with greater use of rapid sequencing technologies in establishing rare genetic disease diagnoses including IEM [9, 14].

In conclusion, we found a high rate of IEM where NBSS and targeted diagnostic evaluation were used in tandem. Despite this, further optimisation of the diagnostic process is possible, including set up of consultation services with paediatric metabolic services and metabolic laboratories to allow for targeted metabolic testing in sick neonates. The diagnostic work-up may have to be repeated and expanded, depending on clinical course and diagnostic findings, as IEM can present at any age and phenotypes may also vary. Where first line metabolic investigations are unrevealing, the diagnostic pathway may be assisted with the use of newer genomic technologies.

## Data Availability

Data will be made available on request.

## Abbreviations

GA: Glutaric aciduria type 1
GAGs: Glycosaminoglycans
GALT: Galactose-1-phosphate uridyltransferase
GC-MS: Gas chromatography mass spectrometry
GIR: Glucose infusion rate
HCU: Classical homocystinuria
IEM: Inborn error of metabolism
LC–MS/MS: Liquid chromatography tandem mass spectrometry
MCADD: Medium-chain acyl-CoA dehydrogenase deficiency
MSUD: Maple syrup urine disease
NBSS: Newborn bloodspot screening
NICU: Neonatal intensive care unit
NSIGHT: Newborn Sequencing in Genomic Medicine and Public Health
PICU: Paediatric intensive care unit
PKU: Phenylketonuria
TPN: Total parenteral nutrition
UCD: Urea cycle defect
